# Retrospective analysis of prognostic factors in patients of papillary thyroid microcarcinoma

**DOI:** 10.18632/oncotarget.26248

**Published:** 2018-10-30

**Authors:** Tinghai Xiang, Wenyan Yan, Longan Zhou

**Affiliations:** ^1^ Department of General Surgery, Binzhou People’s Hospital, Binzhou 256610, Shandong, China; ^2^ Section II, Department of Neurology, Binzhou People’s Hospital, Binzhou 256610, Shandong, China

**Keywords:** papillary thyroid microcarcinoma, central lymph node dissection, therapy, prognosis

## Abstract

We performed a retrospective chart review of 245 patients with papillary thyroid microcarcinoma (PTMC) to define factors linked to central lymph node metastasis and thus prognosis. Univariate and multivariate analyses showed that being male (*p* < 0.001), age <45 years at diagnosis (*p* = 0.045), maximum tumor size > 5 mm (*p* = 0.030), multifocal tumor (*p* = 0.040) and tumor envelope invasion (*p* < 0.001) were all independent risk factors for central compartment lymph node metastasis. Unifocal lesions at the thyroid gland’s upper pole, middle and lower pole, had lymph node metastasis rates of 22.7%, 14.0% and 35.0%, respectively (*p* = 0.048). The rate of central lymph node metastasis was much higher when there was bilateral thyroid involvement than with multifocal unilateral lesions (58.6% vs 37.5%; *p* = 0.040). These results suggest that for patients at low risk of central lymph node metastasis, unilateral thyroid lobe and isthmus resection is sufficient. However, for patients at high risk of central lymph node metastasis, central lymph node dissection increases the likelihood of complete tumor excision.

## INTRODUCTION

Papillary thyroid microcarcinomas (PTMCs) are small papillary thyroid carcinomas with maximum diameters less than 1.0 cm. Cervical lymph node metastasis is the most frequent cause of long-term recurrence, with a rate of about 12% [1]. Although the overall prognosis of PRMC patients is good, some PTMCs are prone to glandular invasion and lymph node metastasis. Consequently, if the treatment is not complete, the risk of recurrence can be as high as 20–30% and ultimately affect the patient prognosis [2, 3]. In the present study, we retrospectively reviewed the surgical treatment of PTMC and analyzed factors that could affect prognosis, including metastasis of central compartment lymph nodes.

## RESULTS

### Univariate analysis of cervical lymph node metastasis

Univariate analyses showed that the risk factors for cervical lymph node metastasis in PTMC patients included being male, the diagnosed at <45 years of age, maximum tumor maximum size >5 mm, multifocal tumor, and tumor envelope invasion (Table 1). Hashimoto’s thyroiditis was not a risk factor for cervical lymph node metastasis.

**Table 1:** Univariate analyses of central lymph node metastasis

Parameters	CLNM(−)	CLNM(+)	Metastasis rate	χ^2^	*p*
	(*n* = 159)	(*n* = 86)	(%)		
Gender				27.570	0.000
Male	45	54	54.5		
Female	114	32	21.9		
Age (years)				4.447	0.035
<45	68	25	26.9		
≥45	91	61	40.1		
Tumor size (mm)				4.853	0.028
>5	88	60	40.5		
≤5	71	26	26.8		
Capsular invasion				20.133	0.000
Yes	38	45	54.2		
No	121	41	25.3		
PTMC with HT				0.399	0.528
Yes	37	17	31.5		
No	122	62	33.7		
Tumor location					
Single focus				6.090	0.048
Upper pole	34	10	22.7		
Middle third	37	6	14.0		
Lower pole	39	21	35.0		
Multifocal				4.224	0.040
Unilateral	25	15	37.5		
Bilateral	24	34	58.6		

### Multivariate analysis of cervical lymph node metastasis

We next performed a multivariate analysis using the significant indicators from the univariate analyses with a two-classification logistic regression model. This analysis showed that diagnosis of PTMC at <45 years of age (*p* = 0.045), being male (*p* < 0.001), maximum tumor diameter >5 mm (*p* = 0.030), and tumor envelope invasion (*P* < 0.001) were all independent risk factors for cervical lymph node metastasis. Our findings also indicated that the lymph node metastasis rates for single lesions located at the upper pole, middle and lower pole of the thyroid gland (22.7%, 14.0% and 35.0%, respectively) differ significantly (*p* < 0.05). When the tumor was located in the bilateral thyroid glands, the rate of central lymph node metastasis was much higher than with multifocal unilateral lesions (58.6% vs 37.5%; *p* = 0.040) (Table 2).

**Table 2:** Multivariate logistic regression analysis of central lymph node metastasis

Parameters	B	S.E.	Wald	df	*p*	95% CL
Gender	0.634	0.142	27.014	1	0.000	1.230∼2.385
Age	−0.501	0.099	3.185	1	0.045	0.426∼1.018
Tumor size	0.836	0.126	4.015	1	0.030	0.363∼0.658
Capsular invasion	−0.510	0.143	18.135	1	0.000	0.524∼0.897
Multifocal			1.988	1	0.040	
1	0.162	0.186	0.902	1	0.080	0.598∼0.721
≥2	0.575	0.301	0.719	1	0.010	0.951∼1.827
Bilateral	0.461	0.197	4.250	1	0.021	1.321∼2.459
Constant	−0.523	0.419	0.234	1	0.459	—

### Relationship between numbers of lesions and central lymph node metastasis

Sixty percent of patients presented with a single tumor, while 40% had 2 or more tumors. Single factor analysis showed that the number of tumors was related to lymph node metastasis (*p* = 0.040), and that the lymph node metastasis was nearly twice as likely to occur in patients with multifocal tumors (50.0% vs 25.2%; *p* = 0.021) (Table 1).

## DISCUSSION

### The prognosis of patients with PTMC

The detection rate of PTMC has been steadily increasing thanks to improvements in diagnostic ultrasound technology and the wide application of ultrasound-guided fine needle aspiration biopsy [4, 5]. Although it was generally believed that the majority of PTMCs progress slowly and that patient prognosis is good, some patients exhibit central, and even lateral, cervical lymph node metastasis at the time of surgical treatment. The reported central lymph node metastasis rate in PTMC ranges from 24.1% to 64.1% [6–8]. In our study, central lymph node metastasis was detected in 35.1% of cases. Single and multiple factor regression analyses indicated that being male, age >45 years, tumor capsule/gland external invasion, and diameter >5 mm are all risk factors for lymph node metastasis in these PTMC patients, which is consistent with earlier reports [9–11].

At present, the relationship between lesion location and lymph node metastasis remains controversial. The presence of a unifocal PTMC at the lower pole of the thyroid gland increases the likelihood of central compartment lymph node metastasis. In cases of multifocal PTMC, the rate of metastasis to central region lymph nodes is lower when only one side of the thyroid is involved. However, the current consensus is that multifocal PTMCs present a high risk for lymph node metastasis [12, 13].

### Treatment of PTMC

There was still no consensus as to whether preventive CLND should be a routine part of PTMC treatment. Whereas routine CLND in “not recommended” in the 2015 edition of the American Thyroid Association management guidelines [14], 2016 guidelines for the diagnosis and treatment of PTMC from the Chinese Cancer Society advise “routine prophylactic CLND under the premise of technical support” [12].

As metastatic central compartment lymph nodes are usually small, the clavicle and manubrium sterni often prevent satisfactory detection of metastatic nodes using ultrasound [15, 16]. In the present study, the sensitivities of ultrasound and intraoperative diagnosis was low, and even when the two methods are combined the sensitivity was only 32%, which is not sufficient to guide the surgeons as to extent of operation. Central lymph node metastasis can readily progress to invasion of the recurrent laryngeal nerve, trachea or esophagus, and even the carotid common artery, which reduces curative potential of operation. The most common occurrence of local residual tumor is in the central region [17]. This makes it a tricky task for even the most experienced surgeon to protect the parathyroid and recurrent laryngeal nerve during the second surgery.

For decades the standard treatment for PTMC has been total thyroidectomy with postoperative radioactive iodine (RAI) treatment. For PTMC invading structures outside the thyroid gland, cervical lymph node metastasis or multiple lesions, postoperative RAI treatment did not reduce the recurrence rate [18, 19]. RAI may cause temporary or permanent damage to the salivary and lacrimal glands and increase the risk of secondary carcinoma in patients [20]. For low-risk patients with metastasis of central compartment lymph nodes, unilateral thyroidectomy thought to be was the best approach [21]. In a large-scale study of 23,605 patients with differentiated thyroid cancer, the total survival rate did not significantly differ between total and unilateral thyroidectomy, but CLND can significantly reduce disease-free survival [22]. In one large study, lymph node metastasis was predictive of adverse outcomes, but the 14-year survival rates were 82% and 79%, respectively, among patients without and with lymph node metastasis [23]. This suggests CLND improves the survival rate among PTMC patients with central lymph node metastasis. In the present study, there were no instances of distant metastasis or death among patients at low risk of central lymph node metastasis treated with unilateral thyroid lobe and isthmus resection, suggesting that approach is sufficient in such cases. For patients at high-risk of central lymph node metastasis, we suggest resection of the thyroid lobe and simultaneous ipsilateral CLND.

The main reason not to recommend simultaneous CLND is the potential increase in postoperative complications, especially injury to the parathyroid gland and recurrent laryngeal nerve during secondary operations. With total thyroidectomy and bilateral CLND, there is an increased risk of parathyroid injury and laryngeal recurrent nerve palsy. On the other hand, with careful operative procedures, including the use of new techniques such as nanocarbon staining, there need not be a significant increase in the incidence of postoperative complications [24].

## PATIENTS AND METHODS

### Patient demographics

We collected and retrospectively analyzed the clinical data from 245 patients with PTMC treated between January 2011 and December 2016 in the Department of General Surgery, Binzhou People’s Hospital. The criteria for inclusion in the study were: (1) initial surgery for thyroid cancer; (2) postoperative pathology confirming the lesion to be PTMC; (3) unilateral or total thyroidectomy with simultaneous central lymph node excision; and (4) availability of complete clinical and pathological data. Exclusion criteria were: (1) PTMC with distant metastasis: (2) other types of thyroid cancer, such as medullary cancer; and (3) carcinoma of more 1.0 cm diameter within multifocal PTMC.

A total of 245 cases were included in this study, including 99 males and 146 females. The average age was 48.5 ± 9.8 years (range: 20–81 years), with 93 patients <45 years old (38.0%) and 152 patients ≥45 years old (62.0%). The average tumor size was 0.57 ± 0.27 cm. There were 147 cases (60.0%) of single focus PTMC and 98 cases (40.0%) of multifocal PTMC, among which there were 58 cases (23.3%) with bilateral tumors. There were 83 cases (33.9%) with invasion of the glandular membrane and 86 cases (35.1%) with central lymph node metastasis (Table 3).

**Table 3:** Clinicopathological characteristics of patients with papillary thyroid microcarcinoma

Parameters	Cases	%
Gender		
Male	99	40.4
Female	146	59.6
Age (years)		
<45	93	38.0
≥45	152	62.0
Tumor size (mm)		
>5	148	60.4
≤5	97	39.6
Capsular invasion		
Yes	83	33.9
No	162	66.1
PTMC with HT		
Yes	54	22.0
No	184	78.0
Multifocal		
Yes	98	40.0
No	147	60.0
Bilateralism		
Yes	58	23.6
No	187	76.4
CLNM		
Yes	59	24.1
No	186	75.9

Abbreviations: PTMC: papillary thyroid microcarcinoma; HT: Hashimoto’s thyroiditis; CLNM: central lymph node metastasis.

### Treatment protocols

All PTMC cases were treated surgically. When nodules were in the unilateral thyroid, only the affected thyroid lobe, its isthmus, and the ipsilateral central lymph nodes were resected. When nodules were detected in both sides of the thyroid gland, total thyroidectomy with bilateral resection of central lymph nodes was performed.

According to the 2002 edition of the American Academy of Otolaryngology-Head and Neck Surgery [25], the extent of central lymph node dissection (CLND) should be as follows: the upper boundary is the lower margin of the hyoid bone; the lower boundary reaches the sternum; the outside edge is the medial carotid sheath, including the near or distal tracheal lymph node and the anterior laryngeal lymph node. Transient hypocalcemia (blood calcium < 2.0 mmo1/L) was defined as present only within 24 h after surgery. Thirty minutes after injecting nanocarbon into the thyroid, the central region lymph nodes and thyroid gland stained black were carefully dissected so as to protect the parathyroid. Thyrotropic hormone inhibitor was administered postoperatively to all patients.

### Statistical analysis

Continuous and normally distributed variables are presented as the mean ± standard deviation. Univariate analyses were done using the chi-square test, while logistic regression was used for multivariate analysis. All analyses were performed using PASW statistics 18.0 software. Values of *p* < 0.05 were considered significant.
